# Fast eating is a strong risk factor for new-onset diabetes among the Japanese general population

**DOI:** 10.1038/s41598-019-44477-9

**Published:** 2019-06-03

**Authors:** Akihiro Kudo, Koichi Asahi, Hiroaki Satoh, Kunitoshi Iseki, Toshiki Moriyama, Kunihiro Yamagata, Kazuhiko Tsuruya, Shouichi Fujimoto, Ichiei Narita, Tsuneo Konta, Masahide Kondo, Yugo Shibagaki, Masato Kasahara, Tsuyoshi Watanabe, Michio Shimabukuro

**Affiliations:** 1 Department of Diabetes, Endocrinology and Metabolism, School of Medicine, Fukushima Medical University, Fukushima, Japan; 2 Steering Committee of Research on Design of the Comprehensive Health Care System for Chronic Kidney Disease (CKD) Based on the Individual Risk Assessment by Specific Health Check, Fukushima, Japan

**Keywords:** Type 2 diabetes, Risk factors

## Abstract

Although many studies that have examined the relationship of type and amount of food and the frequency of eating with new onset of diabetes, there are few reports on the relationship between how meals are eaten, such as skipping breakfast, snacking or food ingestion speed, and the onset of diabetes. We investigated the relationship between eating speed, as well as other eating habits such as snacking and skip breakfast, and new onset of diabetes in a nation-wide Japanese cohort. We obtained data from the nation-wide annual health check program in Japan. In 197,825 participants without diabetes in 2008, questionnaires recorded data on the diet habits (eating speed, snack after supper or before sleep, and skipping breakfast) and unadjusted and multivariable-adjusted logistic regression models were used to measure the odds ratio of new-onset diabetes mellitus in a 3-year follow up. The proportion of fast eaters, those who snack after supper, snack before sleep, and skip breakfast was higher in the new-onset diabetes group than in the group who did not develop diabetes mellitus. As compared with the non-fast eater group, fast eaters were generally younger, had higher BMI, had more weight gain from 20 years onwards, and experienced frequent weight fluctuations of ≥3 kg within 1 year. The risk of fast eaters developing diabetes mellitus remained even after correction for multiple factors including age, body weight, rate of weight change, blood pressure, smoking, and alcohol consumption. No other eating habits were independent predictors for onset of diabetes mellitus. Results show that fast eating is a sole predisposing factor among eating habits for new-onset diabetes. Future studies were warranted to evaluate whether avoidance of fast eating is beneficial for prevention of diabetes mellitus.

## Introduction

Many previous studies clarified the influence of type and amount of food and eating frequency on new onset diabetes^1,2^. There are also reports concerning effects of skipping breakfast and/or snacking on onset of diabetes^3–5^. However, reports on eating speed are scarce. Sakurai *et al*. investigated the association between eating speed and the incidence of type 2 diabetes in 2,050 Japanese men employees of a metal products factory^6^. As compared to slow eating speed, medium (1.68, 95% CI 0.93–3.02) and fast (1.97, 1.10–3.55) eating speed showed increased hazard ratios for new onset of diabetes (p for trend = 0.030). In a Lithuanian case-control study included 234 cases with newly diagnosed type 2 diabetes, fast eaters showed an increased risk of type 2 diabetes as compared to slow eaters (odds ratio 2.52; 95% CI 1.56–4.06, p < 0.001)^7^. However, the assumption is limited by small numbers of subjects, inclusion bias and lack of information for other confounding eating habits.

We investigated the effects of eating speed, as well as other eating habits such as snacking and skip breakfast on new onset of diabetes in a Japanese nation-wide large cohort.

## Results

### General characteristics

General characteristics are shown in Table 1. The average age of the participants was 63.7 years, and 38.0% of participants were men. There were 7032/198187 (3.54%) patients who were not diabetic in 2008 and who developed diabetes between 2009 and 2011. Among fast eaters, the number of patients who developed diabetes mellitus was 30.9% compared with 26.1% who did not develop diabetes mellitus. Among patients who snack after supper, 12.9% developed diabetes mellitus whereas 12.9% did not. Among patients who snack before sleep, 17.2% developed diabetes mellitus compared with 15.0% who did not, and 12.9% of patients who skipped breakfast developed diabetes mellitus compared with 12.4% who did not. Significant differences were found in all categories, and being a fast eater was the most significant risk factor for new-onset diabetes mellitus.

**Table 1:** Clinical characteristics of population at time of enrollment, 2008 and at time of diabetes onset.

Parameters	Total	Diabetes onset − at time of enrollment	Diabetes onset + at time of enrollment	Diabetes onset + at time of diabetes onset	P values*
(n)	197, 825	191, 155	7, 032	7, 032	
Age, years	63.7 (7.7)	63.7 (7.8)	65.2 (6.4)	66.7 (6.4)	<0.01
% Male	38.0	37.5	51.1	51.3	<0.01
BMI, kg/m^2^	22.9 (3.1)	22.8 (3.1)	24.1 (3.5)	24.7 (3.7)	<0.01
Waist circumference, cm	83.2 (8.8)	83.1 (8.7)	86.7 (3.5)	87.5 (9.5)	<0.01
Systolic blood pressure, mmHg	129.5 (17.4)	128.4 (17.4)	133.4 (17.0)	132.2 (16.3)	<0.01
Diastolic blood pressure, mmHg	76.2 (10.6)	76.2 (10.6)	77.8 (10.5)	76.9 (10.6)	<0.01
FPG, mg/dl	93.1 (9.7)	92.8 (9.3)	104.6 (11.6)	118.9 (23.0)	<0.01
HbA1c, %	5.59 (0.33)	5.57 (0.31)	6.01 (0.34)	6.41 (0.65)	<0.01
HDL cholesterol, mg/dL	63.0 (16.1)	63.1 (16.0)	58.5 (15.3)	57.7 (15.1)	<0.01
LDL cholesterol, mg/dL	126.6 (30.0)	126.6 (29.7)	126.4 (31.2)	123.6 (30.6)	0.68
Triglycerides, mg/dL	111.2 (68.4)	110.4 (67.6)	132.2 (88)	134.8 (87.7)	<0.01
AST, U/L	23.9 (9.2)	23.8 (9.1)	25.7 (12.8)	27.2 (15.0)	<0.01
ALT, U/L	21.1 (12.3)	20.9 (12.1)	25.8 (15)	27.6 (19.3)	<0.01
ɤGTP, U/L	34.3(40.1)	33.3 (39.4)	43.5 (52.7)	46.8 (58.8)	<0.01
Hypertension, %	42.4	41.7	60.3	50.9	<0.01
Dyslipidemia, %	53.6	53.2	64.2	32.5	<0.01
Current smoker, %	12.7	12.7	16.6	11.8	<0.01
Eating speed					
Fast, %	26.3	26.1	30.9	30.3	<0.01
Moderate, %	63.4	63.6	59.5	61.1	
Slow, %	10.3	10.3	9.6	8.57	
Non-fast (Moderate + Slow), %	73.7	73.9	69.1	69.7	<0.01
Meal before sleep, %	12.4	12.4	12.9	12.4	<0.01
Snack after supper, %	15.1	15.0	17.2	17.1	<0.01
Breakfast skipping, %	8.1	8.1	8.7	8.7	<0.05
Regular drinking					
Every day, %	22.2	22.1	25.2	22.8	<0.01
Sometimes, %	22.5	22.5	21.9	21.0	
Rarely or none, %	55.3	55.4	53.0	56.1	
Alcohol intake per day					
Under 20 g, %	65.4	65.6	59.4	63.2	<0.01
20 g to less than 40 g, %	24.0	23.9	27.0	24.5	
40 g to less than 60 g, %	8.2	8.1	10.1	9.1	
Over 60 g, %	2.4	2.3	3.5	3.3	
Regular exercise					
Exercise to sweat lightly, %	42.3	42.1	45.6	46.8	<0.01
Walking >1 hour/day, %	52.4	52.4	54.6	56.3	<0.01
Weight Change					
Weight gain over 10 kg from 20-years of age, %	31.1	30.6	46.0	49.0	<0.01
Weight change ± 3 kg within 1 year, %	19.9	19.7	26.9	25.5	<0.01

*Diabetes onset − vs Diabetes onset +.

### Characteristics of fast eaters

We compared the characteristics at baseline for fast eaters with those for moderate + slow eaters (non-fast eaters) (Table 2). Fast eaters were generally younger (61.6 years vs 64.1 years), men (41.6% vs 36.8%), with higher BMI (24.2 vs 22.5 kg/m^2^). Fast eaters more frequently gained weight of >10 kg (40.8% vs 27.7%) and frequent fluctuations of ≥3 kg or more in one year (26.9 vs 17.5%).

**Table 2:** Baseline characteristics of fast and non-fast eating speed groups.

	Fast	Non-fast (Moderate + Slow)	P values*
n	52, 141	146, 046	
Age, years	61.6 (8.5)	64.1 (7.6)	<0.01
% Male	41.6%	36.8%	<0.01
BMI, kg/m^2^	24.2 (3.3)	22.5 (3.0)	<0.01
Waist circumference, cm	85.5 (9.1)	82.5 (8.7)	<0.01
Systolic blood pressure, mmHg	129.1 (17.5)	128.5 (17.4)	
Diastolic blood pressure, mmHg	76.9 (10.9)	76.1 (10.6)	<0.01
FPG, mg/dl	94.2 (12.1)	92.9 (10.3)	<0.01
HbA1c, %	5.59 (0.33)	5.58 (0.33)	<0.01
HDL cholesterol, mg/dL	61.3 (15.7)	63.7 (16.2)	<0.01
LDL cholesterol, mg/dL	127.2 (30.0)	126.4 (30.0)	0.05
Triglycerides, mg/dL	117.0(72.6)	109.1 (67.0)	<0.01
AST,U/L	23.0 (9.4)	23.9 (9.2)	0.05
ALT, U/L	22.2 (13.6)	20.6 (11.7)	<0.01
ɤGTP, U/L	34.8 (40.2)	33.3 (40.0)	<0.01
Hypertension, %	44.2	41.7	<0.01
Dyslipidemia, %	56.4	52.6	<0.01
Current smoker, %	13.9	12.4	<0.01
Snack after supper	18.3	13.9	<0.01
Snack before sleep	16.9	10.8	<0.01
Breakfast skipping	10.2	7.3	<0.01
Regular drinking			
Every day, %	22.8	21.9	<0.01
Sometimes, %	23.1	22.3	
Rarely or none, %	54.1	55.8	
Alcohol intake per day			
Under 20 g, %	62.6	66.4	<0.01
20 g to less than 40 g, %	24.6	23.8	
40 g to less than 60 g, %	9.3	7.7	
Over 60 g, %	3.4	2.0	
Regular exercise			
Exercise to seat lightly, %	42.4	42.2	0.20
Walking >1 hour/day, %	52.6	52.4	<0.01
Weight change			
Weight gain over 10 kg from 20-years of age,%	40.8	27.7	<0.01
Weight change ± 3 kg within 1 year, %	26.9	17.5	<0.01

*Fast vs Non-fast.

At baseline, in men, the average BMI in fast eaters 24.1 kg/m^2^ vs 23.4 kg/m^2^ in non-fast eaters indicate + BMI 0.7 kg/m^2^ difference (P < 0.01). In women, the average BMI in fast eaters 23.4 kg/m^2^ vs 22.2 kg/m^2^ in non-fast eaters indicate + BMI 1.2 kg/m^2^ (*P* < 0.01).

### Odds ratio of new diabetes onset by logistic regression analysis

The odds ratio (OR) for onset of diabetes due to difference in eating habits was examined by logistic regression analysis. Being a fast eater les to a significantly increased risk of diabetes mellitus (OR 1.26, 95% confidence interval [CI] 1.20–1.33, P < 0.01), as did snacking before sleep (OR 1.17, 95% CI 1.10–1.25, P < 0.01), and skipping breakfast (OR 1.09, 95% CI 1.00–1.19, P < 0.05) (Model 1). In Model 3, snacking before sleep and skipping breakfast had no significant differences. There was no difference in patients who snacked after supper with regard to unadjusted OR; however, it was identified as significant following multivariate risk correction (Model 3). In Model 4, even if “change in body weight from 20 years old was 10 kg or more” and “a change in weight over 1 year of ≥3 kg” were included as adjustment factors, fast eaters was an independent factor for diabetes onset. Snacking before sleep, snacking after supper, and skipping breakfast were not independent factors.

Because alcohol drinking might be mutually correlated with eating habits, we compiled degree of the alcohol consumption or presence of regular drinking on the OR calculation as follows. In the model compiling alcohol intake per day (Model 5). Results indicated that fast eating and alcohol consumption was independently and inversely associated with diabetes onset. In the model compiling presence or absence of regular (everyday) drinking (Model 6). Results indicated that fast eating and regular (everyday) drinking was independently and inversely associated with diabetes onset.

## Discussion

This study obtained three major findings. First, the proportion of fast eaters, those who snack after supper, snack before sleep, and skip breakfast was higher in the new-onset diabetes group than in the group who did not develop diabetes mellitus (Table 1). Second, the fast eaters were younger, had higher BMI, had more weight gain from 20 years onwards, and experienced frequent weight fluctuations of ≥3 kg within 1 year as compared with the non-fast eater group (Table 2). Third, the risk of fast eaters developing diabetes mellitus remained significant after correction for multiple factors including age, body weight, rate of weight change, blood pressure, smoking, and alcohol consumption. Other major eating habits were not independent predictors for onset of diabetes mellitus. This study showed for the first time that fast eating is a sole predisposing factor among major eating habits for new-onset diabetes in a large cohort.

### Eating habits and onset of diabetes mellitus

It is well known that breakfast skipping and frequent snacking are related to onset of diabetes with an increase in obesity^3–5^. In our cohort, fast eaters were prevalent in the group of diabetes onset as well as those who snack after supper and before sleep, and skip breakfast (Table 1). Agreed to earlier reports^6,7^, our large cohort study showed that fast eating is a determinant of new-onset diabetes mellitus. We further clarified the effects of eating speed were observed independently of obesity and other diabetes-prone eating habits.

### Characteristics of fast eaters

The fast eaters in our population were younger men with higher BMI and waist circumference, lower HDL and higher triglycerides. Ohkuma *et al*. examined the relationship between eating speed and BMI in a meta-analysis of 15 observational studies, showing that BMI is higher in fast eaters by an average of 1.78 kg/m^2^ (95% confidence interval [CI], 1.53–2.04 kg/m^2^) as compared to slow eaters^8^. Our fast eaters also showed +1.70 kg/m^2^ BMI as compared to non-fast eaters at baseline. On the other hand, fast eaters also showed a higher weight gain. In longitudinal studies, body weight gains were: fast eaters+4.49 kg vs non-fast eaters +3.08 kg after 7 years) in USA fire service personnel^9^ and fast eaters +1.9 kg vs medium and slow eaters +0.7 kg after 8 years in a Japanese company health-checkup^10^. In our study, fast eaters vs non-fast eaters average weight gains from 2008 to 2011 were comparable in overall participants (−0.13 kg vs −0.20 kg, not significant). However, when fast eaters were divided into diabetes onset+ and onset−, the change in body weight was statistically significant: in the diabetes onset group, body weight was 63.7 kg at baseline vs 64.8 kg at onset (+1.1 kg, *P* < 0.01); in the non-onset group, body weight was 60.2 kg at baseline vs 60.0 kg at last observation (2011) (−0.2 kg, *P* < 0.01).

### Fast eaters and new-onset diabetes

Until now, information about the relationship between eating speed and onset of diabetes has been limited^6,7^. Radzeviciene showed that the OR of fast eaters for new-onset diabetes was 2.52 among 234 Lithuanian with new-onset diabetes cases and 468 non-diabetic controls^7^. Sakurai *et al*. reported that the OR of fast eaters was 1.97 (1.10–3.55) compared with slow eaters in Japanese men employees with an average age of 45.9 years (35–55). However, when adjusted for BMI, there was no significant difference^6^.

**Figure 1:**
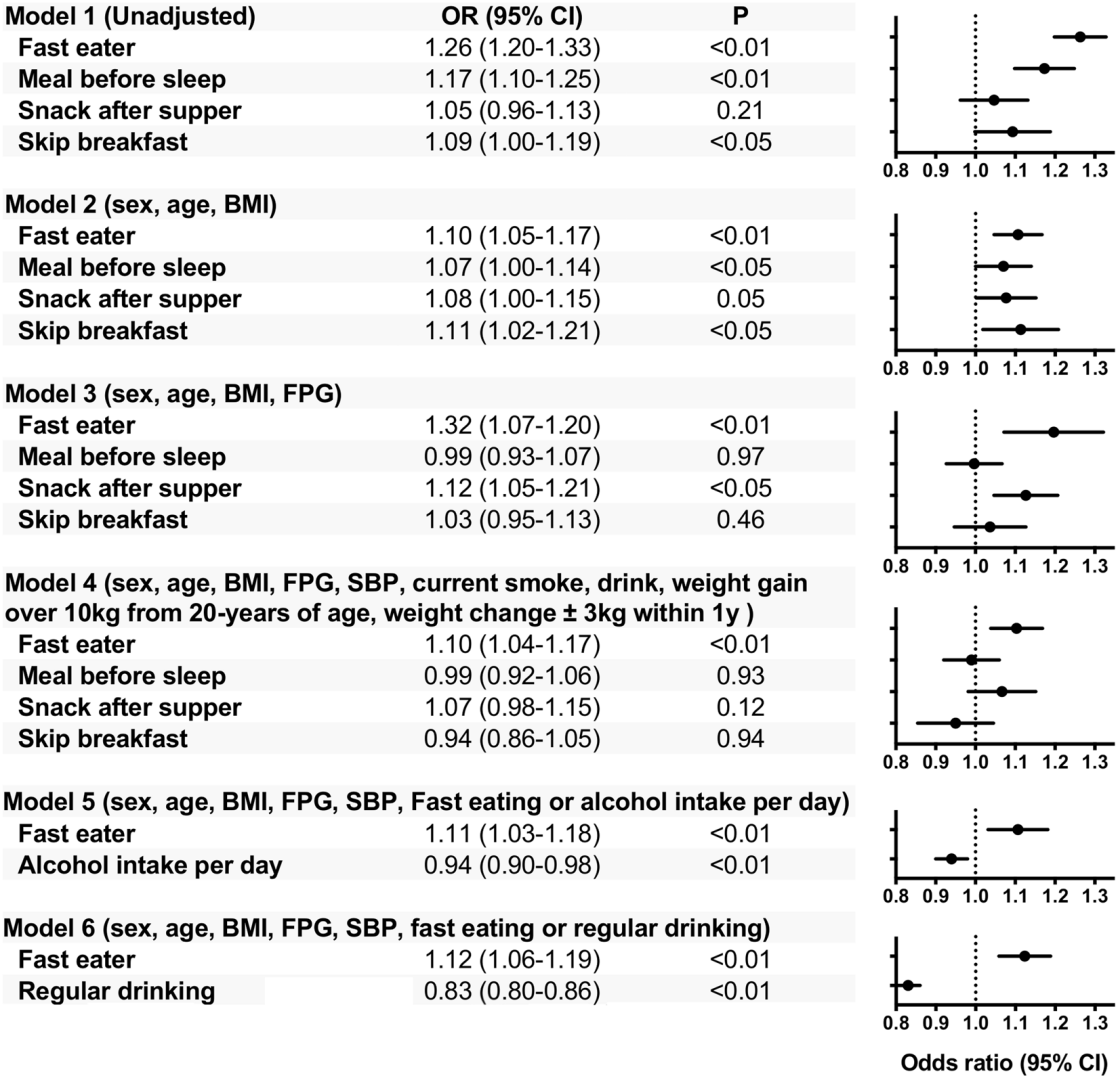
Odds ratio of new diabetes onset by logistic regression analysis Among participants who underwent Japanese nation-wide annual health check program in 2008, non-diabetic participants (n = 197, 825), between the age of 40 and 74 years, were selected and questionnaires recorded data on the diet habits (eating speed, snack after supper or before sleep, and skipping breakfast) and unadjusted and multivariable-adjusted logistic regression models were used to measure the odds ratio of new-onset diabetes mellitus in a 3-year follow up. Unadjusted (Model 1) and adjusted (Model 2–6) odds ratios are shown as closed circles and dotted lines (95% confidential intervals). See details in the methods.

This study showed that fast eating is a sole independent determinant among major eating habits such as snacking before sleep, snacking after supper, and skipping breakfast for the development of diabetes mellitus. It has been reported that late-night snacks, snacks, skipping breakfast, or overeating are independent factors for diabetes onset^4,5^. However, in this large-scale study with heterogenous populations in terms of gender, age, area and jobs, there was no significant difference when correction was made for confounding factors including snacking before sleep, snacking after supper, or skipping breakfast (Fig. 1, Models 3, 4), suggesting that fast eaters are exceptionally in a high-risk group for the development of new-onset diabetes.

### Possible mechanism of diabetes onset by fast eating

Although the mechanism of diabetes onset in fast eaters cannot be elucidated in the present study, previous studies have suggested the underlying mechanism(s). It can be discussed as obesity-dependent and -independent mechanisms.

#### Obesity-dependent mechanisms

It has been reported that workplace stress can increase the likelihood of fast eating^11^. Job stress is often accompanied by a change in eating behaviors including fast eating, eating beyond the point of a full stomach^12^, surrogate meals or overeating, and increased calorie intake^13^, which were speculated in causes of obesity in Japanese men and women^14^. In Japan, men were more likely to be employed on full-time than women; 22.78 million men vs 10.78 million women in full-time employment (http://www.stat.go.jp/english/index.html). There is a large difference in obesity rate between men and women in Japan (BMI ≥ 25.0 kg/m^2^ 28.9% of men vs 17.6% of women); the rate of obesity was also higher among men in our study (men 27.3% vs women 19.9%)^15^. Fast eaters in this study were mainly middle-aged and elderly men engaged in work, where job stress might have led to obesity. The proportion of fast eaters was 28.8% in men and 24.9% in women (Table 3). Taken above, prevalence of fast eaters and obesity can be increased by job stress more habitually in men. The incidence of diabetes was 5.4% among men with fast eating vs 4.5% for total men. The proportion of women fast eaters who developed diabetes was 3.3% vs 2.6% for all women participants. At baseline, BMI was higher in fast eaters both in men and women, but the difference in BMI between fast eaters and non-fast eaters lower in men (+0.7 kg/m^2^) than in women (+1.2 kg/m^2^). Fast eaters in men might be more vulnerable to diabetes even with a small increase in BMI. Effects of eating speed on the risk of obesity and diabetes has also been reported in Japanese men^16^.

**Table 3:** New onset diabetes mellitus of fast and non-fast eating speed groups.

	Total	Male	Female
(n)	197,825	75,186	122,525
Fast (n)	52,141	21,635	30,506
New onset T2DM (n)	2,168	1,158	1,010
Incidence of new onset T2DM (%)	4.2%	5.4%	3.3%
Non-fast (moderate + slow) (n)	145,684	53,551	92,019
New onset T2DM (n)	4,864	2,429	2,420
Incidence of new onset T2DM (%)	3.3%	4.5%	2.6%

T2DM: type 2 diabetes mellitus.

#### Obesity-independent mechanisms

As indicated by Model 4 in Fig. 1, diabetes occurs among fast-eaters irrespective of increases in body weight. First, eating faster reduces energy consumption after meals^17^. Second, when the time for mastication is reduced, glucose and insulin concentrations are significantly higher between 90 and 240 minutes after mastication^18^. Third, in soft-fed rats, postprandial hyperglycemia and hyperinsulinemia decreased levels of IRS2 expression in the liver and Akt phosphorylation was observed, which may lead to increased risk of diabetes^19^. Because alcohol drinking might be mutually correlated with eating habits, we compiled degree of the alcohol consumption or regular drinking on the OR calculation. In the model compiling alcohol or regular (everyday) drinking, fast eater was associated with onset of diabetes independently of the alcohol consumption and presence of regular drinking.

There are limitations to this study. First, in this Japanese nation-wide health examination system, ~51.91 million people aged between 40 and 74 years who should have undergone health examination between March 2008 and April 2009; however, only 20.05 million people (37.4%) underwent the examination and only parts of all participants could be evaluated by our study because of fails of contracts with all local governments. This heterogeneity may bias the results. Second, because of the age limit, the onset of diabetes before age 39 years remains unknown. As such, this analysis is not relevant for juvenile-onset type 2 diabetes. Third, the outcomes based on self-reported data about eating habits. The Questionnaire on eating speed was “How fast do you eat compared to others around same ages? (Faster, Normal, Slower)”. Although the judgement of eating behavior is subjective, this self-reported eating speed has been validated for clinical utility^20^ and can be useful to prevent obesity in the Japanese “Specific Health Check and Guidance System (SHCG)”^21^, supporting our notice. Fourth, the observation period was relatively short. Fifth, our criteria for definition of diabetes, fasting plasma glucose level ≥126 mg/dL or HbA1c levels ≥6.5%, may underestimate onset of diabetes. As reported in a Japanese population^22^, the HbA1c cutoff of 6.0% had appropriate sensitivity and specificity for diabetes screening, suggesting that HbA1c ≥ 6.5% may be inadequate as a screening tool for diabetes.

## Conclusions

In conclusion, fast eating is a sole independent risk factor among major eating habits predisposing to the onset of diabetes. Future studies were warranted to evaluate precise phenotypes of fast eaters and also to evaluate whether avoidance of fast eating is beneficial for prevention of diabetes mellitus.

## Methods

### Participants

The research is not a clinical trial and therefore does not need to be registered. We used data from the annual health check program known as the Japanese “Specific Health Check and Guidance System (SHCG)”^23–26^, launched by the Ministry of Health, Labour and Welfare (MHLW), in 2008. The SHCG aimed to gather data from Japanese people aged between 40 and 74 years; the estimated number of subjects included in the database was 51,919,920. The current study was performed as a part of the ongoing project “Design of the comprehensive healthcare system for chronic kidney disease (CKD) based on the individual risk assessment by Specific Health Checkups”. Twenty-seven of 47 prefectural governments in Japan agreed to participate in this project. One was excluded due to missing follow-up data, leaving 27 prefectures (Hokkaido, Miyagi, Yamagata, Fukushima, Ibaraki, Tochigi, Tokyo, Saitama, Chiba, Kanagawa, Niigata, Nagano, Ishikawa, Fukui, Gifu, Osaka, Hyogo, Okayama, Tokushima, Kochi, Fukuoka, Saga, Nagasaki, Oita, Kumamoto, Miyazaki, and Okinawa). The SHCG data recorded between 2008 and 2011 was sent to and verified by an independent data center - the non-profit organization (NPO) ‘Japan Clinical Support Unit’ (Tokyo, Japan)^23–26^. The community approval was obtained from prefecture representatives. All procedures performed in studies involving human participants were in accordance with the ethical standards of the institutional and/or national research committee at which the studies were conducted (Fukushima Medical University; IRB Approval Number #1485, #2771) and with the 1964 Helsinki declaration and its later amendments or comparable ethical standards. The research was not a clinical trial and therefore did not need to be registered. Our analyses not pre-specified are considered exploratory.

Among 303,654 participants without diabetes mellitus in 2008 (Supplemental Figure), we excluded participants who visited only once in 2008 and those with incomplete data, such as sex, age, body mass index (BMI), systolic blood pressure (SBP), diastolic blood pressure (DBP), fasting plasma glucose levels, glycated hemoglobin (HbA1c). We selected 197,825 participants without diabetes mellitus (see definition below) in 2008.

### Measurements

Trained staff measured the height, body weight, blood pressure, and waist circumference of each subject. Questionnaires recorded data on the following; smoking status (current smoker or not); drinking habits (every day, sometimes, rarely or never); diet habits (eating speed, snack after supper or before sleep, and skipping breakfast); regular exercise (walking >1 hour/day, rarely or never); anti-hypertensive drug use; anti-diabetic drug use; lipid-lowering drug use. Questionnaires about diet habit were: Do you skip breakfast ≥three days per week? (Yes or No), Do you have an evening meal within two hours before bedtime ≥three days per week? (Yes or No), Do you eat a snack after your evening meal (fourth meal) ≥three days per week? (Yes or No), How fast do you eat compared to others around same ages? (Faster, Normal, Slower). Blood samples were collected after an overnight fast and were assayed within 24 hours with automatic clinical chemical analyzers. When required, HbA1c was corrected in line with national glycohemoglobin standardization program equivalent values, calculated using the following formula: HbA1c (%) = HbA1c (Japan Diabetes Society) (%) +0.4% 0.13.

### Definition of diabetes mellitus, hypertension and dyslipidemia

For this study, a participant was considered to have diabetes mellitus when the fasting plasma glucose level was ≥126 mg/dL, when HbA1c levels were ≥6.5% (48 mmol/mol), or if the participant had self-reported the use of anti-hyperglycemic drugs in 2008. Participants were considered to have new-onset diabetes mellitus if they met the above criteria in 2009, 2010, or 2011. Participants were considered hypertensive if their SBP was ≥140 mmHg, if their DBP was ≥90 mmHg, or if they had self-reported the use of antihypertensive drugs. Participants were considered to have dyslipidemia if high-density lipoprotein (HDL)-C levels were <40 mg/dL (1.0 mmol/L), if low-density lipoprotein (LDL)-C levels were ≥140 mg/dL (3.6 mmol/L), if triglyceride (TG) levels were ≥150 mg/dL (1.7 mmol/L), or if they had self-reported the use of lipid-lowering drugs.

### Statistical analyses

A t-test or chi-square test was used to compare the group means (Tables 1 and 2). Unadjusted and multivariable-adjusted logistic regression models were used to estimate the association between new-onset diabetes mellitus and diet habits over 1–3 years of follow-up. In the first step, we carried out unadjusted analyses (Fig. 1, Model 1). In the second step, we adjusted for age, sex, and BMI (Model 2). In the third step, we further adjusted for fasting plasma glucose (Model 3). In the fourth step, we further adjusted for current smoking status, drinking habits, weight gain (over 10 kg from the age of 20 years and weight change ≥3 kg within 1 year) (Model 4). In the fifth steps, model compiling alcohol intake per day as stratified (Model 5): alcohol consumption: 1, under 20 g; 2, 20 g to less than 40 g; 3, 40 g to less than 60 g; 4, over 60 g. In the sixths steps, presence or absence of regular (everyday) drinking (Model 6): 1, rare or none: 2, sometimes; 3, everyday. All analyses were performed using SPSS software (version 24.0; SPSS, Chicago, IL, USA).

### Ethics approval and consent to participate

All procedures performed in studies involving human participants were in accordance with the ethical standards of the institutional and/or national research committee at which the studies were conducted (Fukushima Medical University; IRB Approval Number #1485, #2771) and with the 1964 Helsinki declaration and its later amendments or comparable ethical standards. This study was conducted according also to the Ethical Guidelines for Medical and Health Research Involving Human Subjects enacted by MHLW of Japan [http://www.mhlw.go.jp/file/06-Seisakujouhou-10600000-Daijinkanboukouseikagakuka/0000069410.pdf and http://www.mhlw.go.jp/file/06-Seisakujouhou-10600000-Daijinkanbou-kouseikagakuka/0000080278.pdf].

### Consent for publication

The investigators shall not necessarily be required to obtain informed consent, but we made public information concerning this study on the web [http://www.fmu.ac.jp/univ/sangaku/data/koukai _2/2771.pdf] and ensured the opportunities for the research subjects to refuse utilizing their personal information.

## Supplementary information

Supplemental Figures: Flow chart of participants

## Data Availability

The datasets used and analyzed during the current study are available from the corresponding author on reasonable request.
