# Supplementary material for: Fast eating is a strong risk factor for new-onset diabetes among the Japanese general population
Source: Sci Rep. 2019 Jun 3;9:8210. doi: 10.1038/s41598-019-44477-9 (PMC6547735; doi:10.1038/s41598-019-44477-9)
Supplement: Supplementary file 1 — Supplemental Figures: Flow chart of participants [file 41598_2019_44477_MOESM1_ESM.pdf]

Fast eating is a strong risk factor for new-onset diabetes among the Japanese general population

Akihiro Kudo, Koichi Asahi, Kunitoshi Iseki, Toshiki Moriyama, Kunihiro Yamagata, Kazuhiko Tsuruya, Shouichi Fujimoto, Ichiei Narita, Tsuneo Konta, Masahide Kondo, Yugo Shibagaki, Masato Kasahara, Tsuyoshi Watanabe and Michio Shimabukuro

Online Supplemental Material

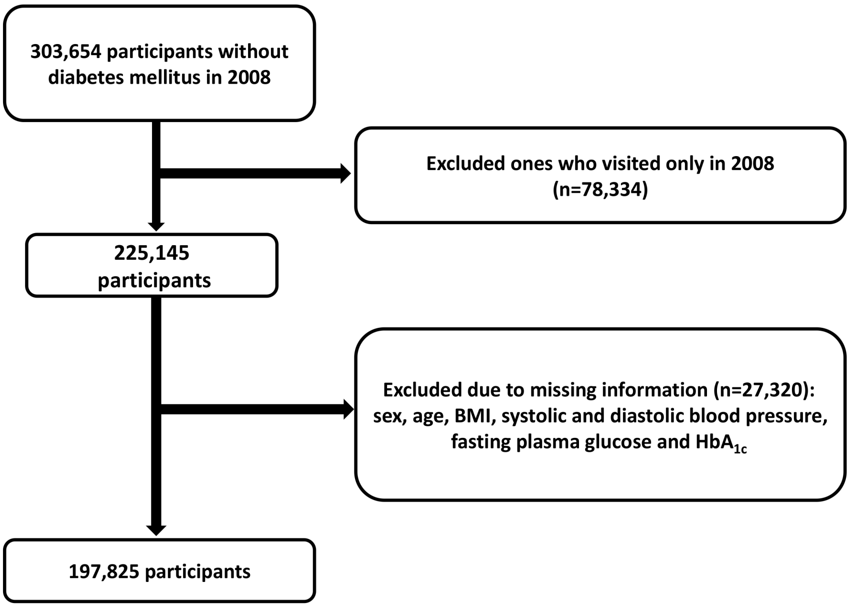

Supplemental Figure. Flowchart of participants.
